# The novel visual cycle inhibitor (±)-RPE65-61 protects retinal photoreceptors from light-induced degeneration

**DOI:** 10.1371/journal.pone.0269437

**Published:** 2022-10-13

**Authors:** Yuhong Wang, Xiang Ma, Parthasarathy Muthuraman, Arun Raja, Aravindan Jayaraman, Konstantin Petrukhin, Christopher L. Cioffi, Jian-Xing Ma, Gennadiy Moiseyev

**Affiliations:** 1 Department of Physiology, University of Oklahoma Health Sciences Center, Oklahoma City, Oklahoma, United States of America; 2 Department of Biochemistry, Wake Forest University School of Medicine, Winston-Salem, North Carolina, United States of America; 3 Department of Basic & Clinical Sciences, Albany College of Pharmacy and Health Sciences, Albany, New York, United States of America; 4 Department of Ophthalmology, Columbia University, New York, New York, United States of America; 5 Department of Chemistry and Chemical Biology, Rensselaer Polytechnic Institute, Troy, New York, United States of America; Universidad de Murcia, SPAIN

## Abstract

The visual cycle refers to a series of biochemical reactions of retinoids in ocular tissues and supports the vision in vertebrates. The visual cycle regenerates visual pigments chromophore, 11-*cis*-retinal, and eliminates its toxic byproducts from the retina, supporting visual function and retinal neuron survival. Unfortunately, during the visual cycle, when 11-*cis*-retinal is being regenerated in the retina, toxic byproducts, such as all-*trans*-retinal and bis-retinoid is N-retinylidene-N-retinylethanolamine (A2E), are produced, which are proposed to contribute to the pathogenesis of the dry form of age-related macular degeneration (AMD). The primary biochemical defect in Stargardt disease (STGD1) is the accelerated synthesis of cytotoxic lipofuscin bisretinoids, such as A2E, in the retinal pigment epithelium (RPE) due to mutations in the *ABCA4* gene. To prevent all-*trans*-retinal—and bisretinoid-mediated retinal degeneration, slowing down the retinoid flow by modulating the visual cycle with a small molecule has been proposed as a therapeutic strategy. The present study describes RPE65-61, a novel, non-retinoid compound, as an inhibitor of RPE65 (a key enzyme in the visual cycle), intended to modulate the excessive activity of the visual cycle to protect the retina from harm degenerative diseases. Our data demonstrated that (±)-**RPE65-61** selectively inhibited retinoid isomerase activity of RPE65, with an IC_50_ of 80 nM. Furthermore, (±)-**RPE65-61** inhibited RPE65 via an uncompetitive mechanism. Systemic administration of (±)-**RPE65-61** in mice resulted in slower chromophore regeneration after light bleach, confirming *in vivo* target engagement and visual cycle modulation. Concomitant protection of the mouse retina from high-intensity light damage was also observed. Furthermore, RPE65-61 down-regulated the cyclic GMP-AMP synthase stimulator of interferon genes (cGAS-STING) pathway, decreased the inflammatory factor, and attenuated retinal apoptosis caused by light-induced retinal damage (LIRD), which led to the preservation of the retinal function. Taken together, (±)-**RPE65-61** is a potent visual cycle modulator that may provide a neuroprotective therapeutic benefit for patients with STGD and AMD.

## Introduction

**Fig 1:**
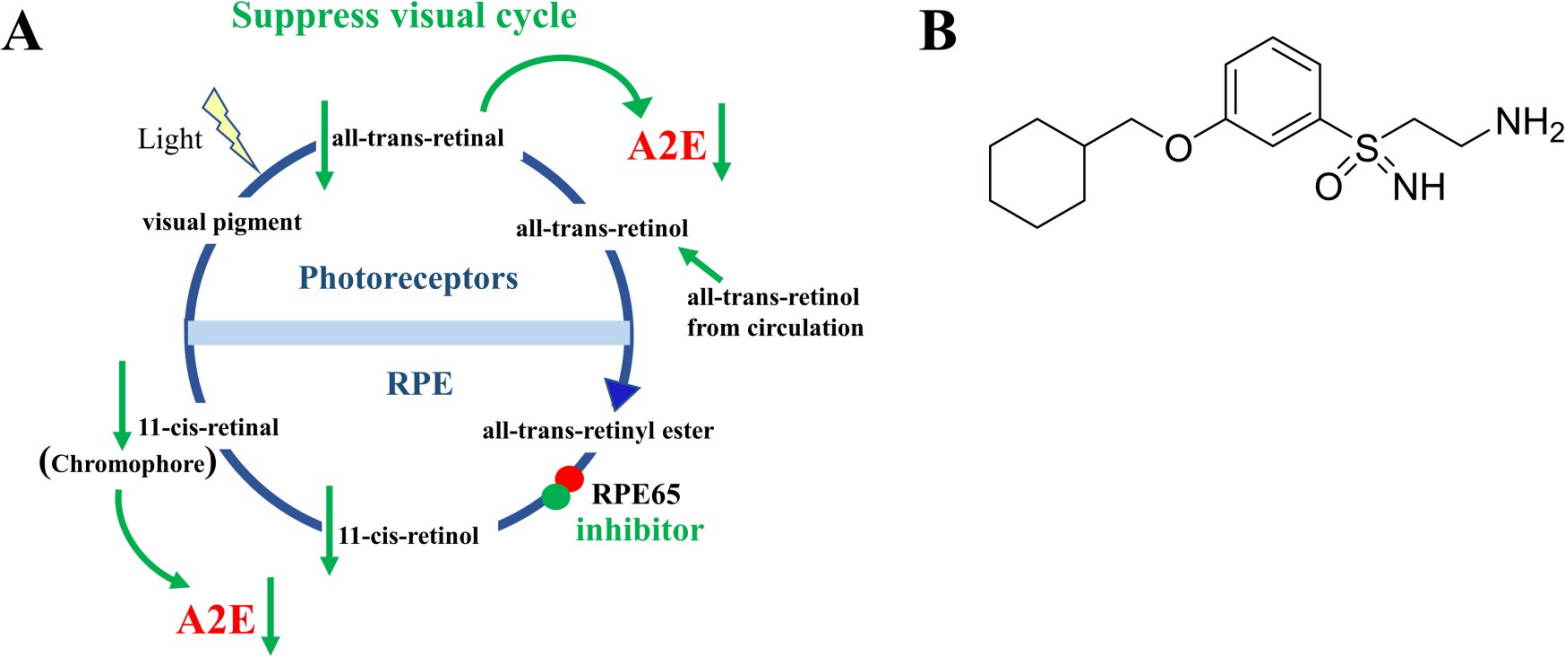
Scheme of visual cycle and structure of inhibitor (±)-RPE65-61. (A) Schematic representation of the visual cycle and the target of (±)-**RPE65-61**. The visual cycle includes a series of biochemical reactions, one of the key steps is the conversion of all-*trans*-retinyl ester to 11-*cis*-retinol by RPE65 isomerase. All-*trans*-retinol converted through a cascade of enzymatic reactions in the retinal pigmented epithelium (RPE), producing a light sensitive chromophore 11-*cis*-retinal, which binds with opsin and allows photon capture in photoreceptor outer segment. All-*trans*-retinal and 11-*cis*-retinal, when accumulated in excessive amounts, can lead to production of A2E which causes oxidative stress and ocular diseases. RPE65-61 selectively inhibits isomerase activity of RPE65 and prevents the retinal damage by slowing down the accumulation of all-*trans*-retinal in light-induced retinal damage animal model. (B) (±)-**RPE65-61** chemical structure.

In vertebrates, both rod and cone visual pigments consist of opsin apo-protein and the chromophore 11-*cis*-retinal. Vision is initiated after absorption of a photon, resulting in photoisomerization of 11-*cis*-retinal to all-*trans*-retinal, which triggers the phototransduction cascade [1]. Regeneration of 11-*cis*-retinal proceeds through the series of biochemical reactions termed the visual cycle [2, 3] (Fig 1). All-*trans*-retinal generated by light absorption is dissociated from opsin and transported out of the lumen of photoreceptor disk membrane to the cytosol via ATP-binding cassette transporter 4 (ABCA4) [4].

Subsequently, all-*trans*-retinal is converted to all-*trans*-retinol by all-*trans*-retinol dehydrogenases [5]. The generated all-*trans*-retinol is then exported from photoreceptors to the retinal pigment epithelium (RPE) through the interphotoreceptor matrix, where it is chaperoned by intracellular retinol-binding protein (IRBP) [6]. In the RPE cells, all-*trans*-retinol is esterified by lecithin retinol acyltransferase (LRAT), producing all-*trans*-retinyl esters [7]. RPE65, a key visual cycle enzyme, converts all-*trans*-retinyl esters to 11-*cis*-retinol [8–10]. The generated 11-*cis*-retinol is oxidized to 11-*cis*-retinal by 11-*cis*-retinol dehydrogenases [11, 12]. 11-*cis*-retinal is then transported back to photoreceptors and recombines with opsin to form visual pigments. As a result of genetic mutations, excessive light, or age, however, a part of all-*trans*-retinal [13] or 11-*cis*-retinal [14] may react with the phosphatidylethanolamine, a component of membranes, producing several bis-retinoids which accumulate in the RPE as a result of photoreceptor phagocytosis. The most studied bis-retinoid is A2E which mediates toxicity in diseases like STGD and AMD [15, 16]. It has also been suggested that an excessive amount of all-*trans*-retinal itself may cause photoreceptor apoptosis [17]. Since bis-retinoids and all-*trans*-retinal formed as byproducts of the visual cycle, a partial inhibition of it was proposed as a dry AMD and STGD treatment plan. The conversion of all-*trans*-retinyl ester to 11-*cis*-retinol catalyzed by RPE65 is a limiting step of the visual cycle [18]. Besides, RPE65 is a unique protein that does not share significant sequence homology to other proteins in humans. All this makes it an attractive drug target to inhibit the visual cycle. Most the well-known RPE65 inhibitors (such as retinylamine and isotretinoin) are retinoids or their analogs with potential off-target activities [19–21]. (*R*)-emixustat, the most thoroughly investigated non-retinoid inhibitor, has several drawbacks, including poor pharmacokinetics (PK) features and signs of retinal toxicities [22, 23]; therefore, the development of new non-retinoid RPE65 inhibitors is of high significance.

This study shows that (±)-**RPE65-61**, a new non-retinoid RPE65 inhibitor, may precisely target RPE65 in vitro and in vivo. We demonstrated that (±)-**RPE65-61** specifically and uncompetitively inhibits RPE65 in vitro. The present study evaluated the inhibitory activity of (±)-**RPE65-61** on 11-*cis*-retinal regeneration in vivo. Furthermore, (±)-**RPE65-61** has a retinal protective function against LIRD, and we propose its molecular mechanism of action.

## Materials and methods

### Animal care

All animal studies were approved by the Institutional Animal Care and Use Committee (IACUC) of the University of Oklahoma Health Sciences Center and performed following the guidelines of the Association for Research in Vision and Ophthalmology (ARVO) statement for the “Use of Animals in Ophthalmic and Vision Research”. BALB/cJ albino mice (2-month-old) with the Rpe65-Leu450 polymorphism were used for all experiments. The animals were maintained from birth in standard housing under dim cyclic light with 12 h light/dark cycles and food and water ad libitum.

### Synthesis of (±)-RPE65-61

All reactions were performed under a dry atmosphere of nitrogen unless otherwise specified. Indicated reaction temperatures refer to the reaction bath, while room temperature (rt) is noted as 25°C. Commercial grade reagents and anhydrous solvents were used as received from vendors and no attempts were made to purify or dry these components further. Removal of solvents under reduced pressure was accomplished with a Buchi rotary evaporator at approximately 28 mm Hg pressure using a Teflon-linked KNF vacuum pump. The measurement of pH for neutralizations or acidifications was measured with Hydrion pH paper (MicroEssential Lab). Thin layer chromatography was performed using 1” x 3” AnalTech No. 02521 silica gel plates with fluorescent indicator. Visualization of TLC plates was made by observation with either short wave UV light (254 nm lamp), 10% phosphomolybdic acid in ethanol or in iodine vapors. Preparative thin layer chromatography was performed using Analtech, 20 × 20 cm, 1000-micron preparative TLC plates. Flash column chromatography was carried out using a Biotage^®^ Selekt System with Teledyne Isco RediSep Rf and Biotage Sfär silica gel columns. If needed, products were purified by reverse phase chromatography, using a Biotage^®^ Selekt System with a RediSep Gold C18 reverse phase column. ^1^H NMR spectra were obtained on a 400 MHz Varian nuclear magnetic resonance spectrometer. Chemical shifts (δ) are reported in parts per million (ppm) and coupling constant (*J*) values are given in Hz, with the following spectral pattern designations: s, singlet; d, doublet; t, triplet, q, quartet; quint, quintet; m, multiplet; dd, doublet of doublets; dt, doublet of triplets; dq; doublet of quartets; br, broad signal. Tetramethylsilane was used as an internal reference. Peak listing, multiplicity designations, and coupling constant calculations were conducted using Mnova v.14 software (Mestrelab Research). ^13^C NMR spectra were obtained on a 500 MHz Bruker AV III nuclear magnetic resonance spectrometer and tetramethylsilane was used as an internal reference. Mass spectroscopic analyses were performed using ESI ionization on a Waters AQUITY UPLC triple quadrupole liquid chromatography mass spectrometer (LCMS). High pressure liquid chromatography (HPLC) purity analysis was performed using a Waters Breeze2 HPLC system with a binary solvent system A and B using a gradient elusion [A, H_2_O with 0.1% formic acid; B, CH_3_CN with 0.1% formic acid] and flow rate = 0.5 mL/min, with UV detection at 254 nm (system equipped with a photodiode array (PDA) detector). An ACQUITY UPLC BEH C18 column, 130 Å, 1.7 μm, 2.1 mm × 50 mm was used. High resolution mass spectrometry (HRMS) analysis was was performed using an Agilent 6530 Accurate-Mass Q-TOF. All final compounds tested for *in vitro* and *in vivo* biological testing were purified to ≥95% purity, and these purity levels were measured by both ^1^H NMR and HPLC.

**Scheme 1:**
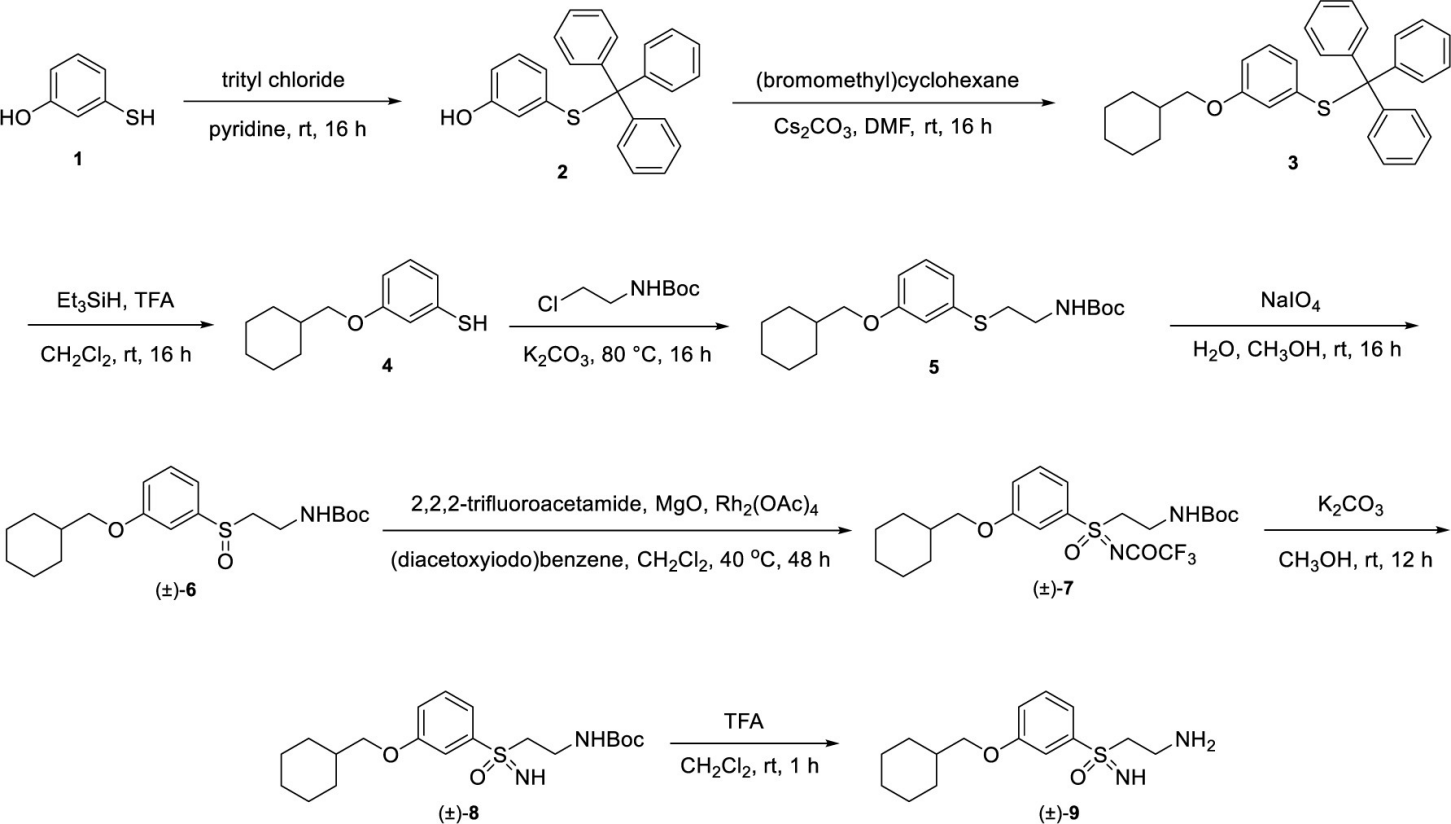
Preparation of (±)-(2-aminoethyl)(3-(cyclohexylmethoxy)phenyl)(imino)-ƛ^6^-sulfanone ((±)-9, (±)-RPE65-61).

The synthesis of (±)-**RPE65-61** is highlighted in Scheme 1 and begins with a trityl protection of 3-mercaptophenol (**1**) to afford phenol **2**. A subsequent Williamson ether synthesis is performed with **2** and (bromomethyl)cyclohexane to yield ether **3**, which undergoes deprotection in the presence of Et_3_SiH in TFA to generate thiol **4**. Alkylation of **4** with *tert*-butyl (2-chloroethyl)carbamate provides thioether **5**, which is oxidized to sulfoxide (±)-**6** with NaIO_4_. Further oxidation of sulfoxide (±)-**6** is conducted to give trifluoroacetamide-substituted sulfoximine (±)-**7**. Removal of the trifluoroacetamide of (±)-**7** is accomplished with K_2_CO_3_ in methanol to afford (±)-**8**, which undergoes TFA-induced Boc de-protection to provide desired (±)-**9** ((±)-**RPE65-61**).

*(±)-(2-aminoethyl)(3-(cyclohexylmethoxy)phenyl)(imino)-ƛ*^*6*^*-sulfanone*. Step A: To a 0°C cooled of 3-mercaptophenol (**1**) (2.00 g, 15.85 mmol) in anhydrous pyridine (20 mL) was added tritylchloride (4.60 g, 16.50 mmol). The resulting mixture stirred at rt under an atmosphere of N_2_ for 16 h. The mixture was then diluted with H_2_O (30 mL) and extracted with CH_2_Cl_2_ (3 × 50 mL). The combined organic extracts were washed with brine, dried over Na_2_SO_4_, filtered and concentrated under reduced pressure to give crude 3-(tritylthio)phenol (**2**) as a brown oil (5.8 g, >99%, crude), which was taken as is into next step without purification.

Step B: To a 0°C cooled solution of 3-(tritylthio)phenol (**2,** 5.8 g, 15.73 mmol) in anhydrous DMF (50 mL) were added Cs_2_CO_3_ (7.60 g, 23.32 mmol) and (bromomethyl)cyclohexane (3.3 g, 18.63 mmol). The resulting mixture stirred at rt under an atmosphere of N_2_ for 16 h. The mixture was then diluted with H_2_O (30 mL) and extracted with EtOAc (3 × 50 mL). The combined organic extracts were washed with brine, dried over Na_2_SO_4_, filtered and concentrated under reduced pressure. The resulting crude residue was chromatographed over silica gel (0–15% EtOAc in hexanes) to give (3-(cyclohexylmethoxy)phenyl)(trityl)sulfane (**3**) as an oil (6.2g, 85%, crude). The material contained an inseparable impurity, which was taken into next step.

Step C: To a 0°C cooled solution of (3-(cyclohexylmethoxy)phenyl)(trityl)sulfane (**3**, 6.2 g, 13.34 mmol) in CH_2_Cl_2_ (30 mL) were added TFA (15 mL, 196 mmol) and Et_3_SiH (6.4 mL g, 40.07 mmol). The resulting mixture stirred at rt under an atmosphere of N_2_ for 16 h. The mixture was concentrated under reduced pressure and the residue was diluted with H_2_O (50 mL). The aqueous mixture was extracted with CH_2_Cl_2_ (3 × 50 mL) and the combined organic extracts were washed with brine, dried over Na_2_SO_4_, filtered and concentrated under reduced pressure. The resulting residue was chromatographed over silica gel (0–5% EtOAc in hexanes) to give 3-(cyclohexylmethoxy)benzenethiol (**4**) as a yellow oil that contained an inseparable impurity (1.5 g, 50%, crude).

Step D: To a solution of 3-(cyclohexylmethoxy)benzenethiol (**4**, 1.5 g, 6.74 mmol) in anhydrous DMF (7 mL) were added Cs_2_CO_3_ (6.60 g, 20.25 mmol) and *tert*-butyl (2-chloroethyl)carbamate (2.4 g, 13.36 mmol) and the resulting mixture was heated at 85°C under an atmosphere of N_2_ for 16 h. The mixture was allowed to cool to rt and then diluted with H_2_O (30 mL). The aqueous mixture was extracted with EtOAc (3 × 50 mL) and the combined organic extracts were washed with brine, dried over Na_2_SO_4_, and concentrated under reduced pressure. The resulting residue was chromatographed over silica gel (0–10% EtOAc in hexanes) to give *tert*-butyl (2-((3-(cyclohexylmethoxy)phenyl)thio)ethyl)carbamate (**5**) as a colorless oil (1.1 g, 45%): ^1^H NMR (400 MHz, acetone-*d*_6_) δ 7.16 (t, *J* = 7.6 Hz, 1H), 6.95 (s, 1H), 6.89 (d, *J* = 7.6 Hz, 1H), 6.71 (d, *J* = 8.4 Hz, 1H), 3.79 (d, *J* = 6.4 Hz, 2H), 3.27–3.22 (m, 2H), 3.02–3.00 (m, 2H), 1.86–1.64 (m, 6H), 1.41 (s, 9H), 1.37–1.18 (m, 4H), 1.01–1.00 (m, 2H); ESI MS *m/z* 297 [M + H]^+^. ESI MS *m/z* 366 [M + H]^+^.

Step E: To a 0°C solution of *tert*-butyl (2-((3-(cyclohexylmethoxy)phenyl)thio)ethyl)carbamate (**5**, 1.2 g, 3.28 mmol) in a mixture of H_2_O (18 mL) and CH_3_OH (12 mL) was added NaIO_4_ (1.05 g, 4.91 mmol). The mixture was heated at 80°C for 16 h then allowed to cool to rt and concentrated under reduced pressure to remove CH_3_OH. The resulting aqueous mixture was extracted with EtOAc (3 × 50 mL) and the combined organic extracts were washed with brine, dried over Na_2_SO_4_, and concentrated under reduced pressure. The resulting residue was chromatographed over silica gel (0–50% EtOAc in hexane) to give (±)-*tert*-butyl (2-((3-(cyclohexylmethoxy)phenyl)sulfinyl)ethyl)carbamate ((±)-**6**) as a white solid (0.580 g, 58%) along with recovered *tert*-butyl (2-((3-(cyclohexylmethoxy)phenyl)thio)ethyl)carbamate (**5**, 0.250 g): ^1^H NMR (400 MHz, acetone-*d*_6_) δ 7.45 (t, *J* = 8 Hz, 1H), 7.22 (s, 1H), 7.17 (d, *J* = 7.6 Hz, 1H), 7.04 (d, *J* = 8.4 Hz, 1H), 3.84 (d, *J* = 6.4 Hz, 2H), 3.49–3.30 (m, 2H), 3.12–3.06 (m, 1H), 2.89–2.83 (m, 1H), 1.86–1.64 (m, 6H), 1.42 (s, 9H), 1.36–1.19 (m, 4H), 1.17–1.04 (m, 2H), ESI MS *m/z* 382 [M + H]^+^.

Step F: To a 0°C cooled solution of *tert*-butyl (2-((3-(cyclohexylmethoxy)phenyl)sulfinyl)ethyl)carbamate (**6**, 0.550 g, 1.44 mmol) in CH_2_Cl_2_ (30 mL) was added 2,2,2-trifluoroacetamide (0.444 g, 3.92 mmol), MgO (0.230 g, 5.71 mmol), PhI(OAc)_2_ (0.695 g, 2.16 mmol) and Rh_2_(OAc)_4_ (31.8 mg, 0.072 mmol). The resulting mixture stirred at rt under an atmosphere of N_2_ for 16 h and was then filtered through Celite. The filtrate was concentrated under reduced pressure and the resulting residue was chromatographed over silica gel (0–60% EtOAc in hexane) to give (±)-*tert*-butyl (2-(3-(cyclohexylmethoxy)-*N*-(2,2,2-trifluoroacetyl)phenylsulfonimidoyl)ethyl)carbamate ((±)-**7**) as a white solid (0.600 g, 85%): ^1^H NMR (400 MHz, acetone-*d*_6_) δ7.63 (t, *J* = 8.4 Hz, 1H), 7.57–7.53 (m, 2H), 7.36 (d, *J* = 8.4 Hz, 1H), 4.00–3.90 (m, 4H), 3.52–3.45 (m, 2H), 1.87–1.65 (m, 6H), 1.33 (s, 9H), 1.27–1.04 (m, 5H), ESI MS *m/z* 493 [M + H]^+^.

Step G: To a stirred solution of (±)-*tert-*butyl (2-(3-(cyclohexylmethoxy)-*N*-(2,2,2-trifluoroacetyl)phenylsulfonimidoyl)ethyl)carbamate as ((±)-**7**, 0.600 g, 1.22 mmol) in CH_3_OH (10 mL) was added K_2_CO_3_ (0.841 g, 6.09 mmol) and the resulting mixture stirred at rt for 12 h. The mixture was concentrated under reduced pressure and the reulting material was taken up in CH_2_Cl_2_ (50 mL). Separation of suspended solid matter was achieved via filteration through Celite. The filter cake was washed with CH_2_Cl_2_ (100 mL) and the filtrate was concentrated under reduced pressure to give crude (±)-*tert*-butyl (2-(3-(cyclohexylmethoxy)phenylsulfonimidoyl)ethyl)carbamate ((±)-**8**) as colorless oil, which was used as is in the next step (0.450 g, 93%): ESI MS *m/z* 397 [M + H]^+^.

Step H: To a 0°C cooled solution of (±)-*tert*-butyl (2-(3-(cyclohexylmethoxy)phenylsulfonimidoyl)ethyl)carbamate ((±)-**8**, 0.450 g 1.13 mmol) in CH_2_Cl_2_ (10 mL) was added TFA (5 mL, 65.33 mmol). The mixture stirred for 1 h at rt and was carefully neutralized via addition of a saturated aqueous solution of NaHCO_3_. The biphasic mixture was separated, and the aqueous layer was further extracted with CH_2_Cl_2_ (3 × 50 mL). The combined organic layers were washed with brine, dried over Na_2_SO_4_, filtered and concentrated under reduced pressure. The resulting residue was chromatographed over silica gel (0–10% CH_3_OH in CH_2_Cl_2_) to give (±)-(2-aminoethyl)(3-(cyclohexylmethoxy)phenyl)(imino)-ƛ^6^-sulfanone ((±)-**9**) as a colorless oil (0.130 g, 39%): ^1^H NMR (400 MHz, DMSO-*d*_6_) δ 7.50 (t, *J* = 8 Hz, 1H), 7.42 (d, *J* = 8 Hz, 1H), 7.34 (s, 1H), 7.20 (d, *J* = 7.2 Hz, 1H), 3.84 (d, *J* = 6 Hz, 2H), 3.24–3.17 (m, 2H), 2.77–2.73 (m, 2H), 2.07–1.63 (m, 6H), 1.26–1.17 (m, 3H), 1.09–1.03 (m, 2H); ^13^C NMR (500 MHz, DMSO-*d*_6_) δ 159.5, 130.8, 120.3, 119.7, 113.9, 73.6, 37.5, 36.1, 29.6, 26.4, 25.7; ESI MS *m/z* 297 [M + H]^+^; HRMS (ESI^+^) C_15_H_24_N_2_O_2_S calcd [M + H]^+^ = 297.1631, observed [M + H]^+^ = 297.1621; combustion analysis (%C,H,N): calcd for C_15_H_24_N_2_O_2_S · 0.7 H_2_O: %C = 58.3; %H = 8.28; %N = 9.06; found: %C = 58.66; %H = 8.02; %N = 8.7; HPLC 98.8% (AUC), *t*_R_ = 12.4 min.

### In vitro retinol isomerase assays

All*-trans* [11,12-^3^H]-retinol (1 mCi/ml, 45.5 Ci/mmol, Perkin Elmer, Boston, MA) in N,N-dimethyl formamide (DMF) was used as the substrate for the isomerase assay. Bovine RPE microsomes were prepared as described previously [24]. For each reaction, 25 μg microsomal proteins from the bovine RPE were added into 200 μl of reaction buffer (10 mM 1,3-bis[tris(hydroxymethyl)-methylamino]propane (BTP), pH 8.0, 100 mM NaCl) containing 0.2 μM of all-*trans* retinol, 1% BSA and 25 μM of cellular retinaldehyde-binding protein (CRALBP). For the inhibition of isomerase activity, (±)-RPE65-61 dissolved in the DMF was added to the reaction prior to addition of all-*trans* retinol. The reaction was stopped, and retinoids extracted with 300 μl of cold methanol and 300 μl of hexane and centrifuged at 10, 000 ×g for 5 min. The upper layer was collected, and the generated retinoids were analyzed by normal phase HPLC as described [24]. The peak of each retinoid isomer was identified based on its characteristic retention time of retinoid standards. The isomerase activity was calculated from the area of the 11-*cis*-retinol peak using Radiomatic 610TR software (Perkin Elmer, Boston, MA) with synthetic 11-*cis* [^3^H]-retinol as a standard. Alternatively, for each reaction, an equal amount of all-*trans* retinyl palmitate (a*t*RP) incorporated into the liposomes (250 μM lipids, 3.3 μM a*t*RP) and 25 μg of purified chicken recombinant RPE65 were incubated in 200 μl of reaction buffer (10 mM BTP, pH 8.0, 100 mM NaCl) containing 0.5% BSA and 25 μM cellular retinaldehyde-binding protein for 2 hr at 37°C. The retinoid profile after completion of the reaction was analyzed by HPLC where the peaks were identified by co-elution with retinoid standards. The RPE65 isomerase activity was calculated based on the 11-*cis*-retinol peak area as described [9, 25]. Nonlinear regression analysis of *v*-versus-[S] data was used to calculate *V*_max_ (apparent) and *K*_m_ (apparent) in the absence and in the presence of the inhibitor (mean ± SEM). The inhibition constant for (±)-RPE65-61 (mean ± SEM) was calculated from the following equation: Ki = [I]/(V_mi_/V_m_ -1), where [I]–concentration of inhibitor, V_m_ is maximal velocity in the absence of the inhibitor, V_mi_ is maximal velocity in the presence of inhibitor [26]. To calculate the standard error, we used nonlinear regression with GraphPad Prism. The initial rate dependences on substrate concentrations were fitted using the uncompetitive inhibition model.

### Quantification of endogenous retinoids using HPLC

Dark-adapted or light-exposed mice were sacrificed, and their eyes enucleated under dim red light. The whole eyes were homogenized with a glass grinder in lysis buffer [10 mM NH_2_OH, 50% ethanol, 50% 2-(N-morpholino) ethanesulfonic acid, pH 6.5], incubated for 1 hr, and retinoids were extracted with hexane. Solvent was evaporated under argon gas, and dried retinoid samples were resuspended in 200 μl of HPLC mobile phase (11.2% ethyl acetate, 2.0% dioxane, 1.4% octanol, 85.4% hexane) and injected into HPLC (515 HPLC pump; Waters Corp., Milford, MA) with a normal phase Lichrosphere SI-60 (Alltech, Deerfield, IL) 5 μm column and isocratic mobile phase (1 ml/min) [27].

### Light-induced retinal damage (LIRD)

Mice were dark-adapted overnight with food and water ad libitum. On the following day, mice were intraperitoneally injected with freshly prepared (±)-RPE65-61 or vehicle (Solutol HS 15 (BASF Corp, Florham Park, NJ, USA) and dimethyl sulfoxide (DMSO) dissolved in sterile saline (0.9% NaCl)). One hour after the systemic administration of (±)-RPE65-61 or vehicle, mice were placed in a light box illuminated with white fluorescent tube lights (10,000 lux for 3 hr). The mice were returned to regular housing for 5 days and then the retinal damage was assessed. As a model, BALB/cJ mice exhibit significantly higher levels of endogenous activity of RPE65 relative to other mouse strains [24]. The impact of the visual cycle inhibitor may therefore be monitored more precisely.

### Histology

The superior side of the cornea was demarcated with green tattoo dye, and the eyes were carefully enucleated. The eyes were immersed in Prefer’s fixative (manufactured by Anatech Ltd) for 30 min and kept in 70% ethanol until they were embedded in paraffin. Sagittal sections along the superior-inferior retinal axis were cut, and the slides were deparaffinized prior to hematoxylin and eosin staining. Light microscopy was performed with Olympus Provis Ax-70 microscope, and the acquired images were analyzed with Image J software (NIH, Bethesda) [28].

### Effect of inhibitor ((±)-RPE65-61 on retinal apoptosis

To further evaluate the effect of (±)-**RPE65-61** on retinal apoptosis, retinal sections from LIRD mice were used for TUNEL with the In Situ Cell Death Detection Kit (Millipore/Sigma Cat#:11684795910). TUNEL-positive photoreceptor cells were quantified to evaluate the effects of (±)-RPE65-61 as described [29].

### OCT and quantification of retinal thickness

Spectral-domain (SD) -OCT device (Bioptigen Inc. Durham. NC, USA) was used to record the retinal layers thickness [30, 31]. Images were captured with the rectangular scan at 1000 A-scans per B-scan, and 100 B-scans per frame. Total retinal thickness (TRT) was measured perpendicularly from the layer of retinal nerve fiber layer (RNFL) to the layer of outer segment (OS), 500 μm away from the center of optic nerve head (ONH) using the built-in software (InVivoVU, Bioptigen) and then averaged. The examiners were blinded to the treatment information.

### ERG recording

Mice were dark-adapted overnight prior to ERG recording. Mice were anesthetized by an intraperitoneal injection of 2 μl/g body weight of 40 mg/ml ketamine and 3 mg/ml xylazine diluted with saline. Pupils were dilated with 1% cyclopentolate hydrochloride ophthalmic solution (Cyclogyl) (Sandoz Inc, Princeton, NJ) and 10% phenylephrine-HCl. Hypromellose ophthalmic demulcent solution (Goniovisc; 2.5%) (HUB pharmaceuticals LLC, Rancho Cucamonga, CA) was applied to each cornea, followed by the placement of gold wire electrodes. Stainless steel electrodes were placed into the right cheek and the tail to serve as a reference and the ground, respectively. Dark-adaptation recovery protocol was run using an Espion E3 system with a Ganzfeld ColorDome system (Diagnosys LLC, Lowell, MA) [28]. The retinal function was measured with ERG using Espion E3 system Ganzfeld Color Dome system (Diagnosys). The Scotopic ERG was recorded using a series of flashes with increasing light intensities (from 0.002 to 400 cd·s/m2). Photopic ERG was performed after the mice were light-adapted in 50 cd/m2 background dome for 10 min. Photopic ERG was recorded with a 2000 cd·s/m2 flash.

### Statistical analyses

GraphPad Prism 8.0 software (GraphPad Software, Inc., La Jolla, CA) was used for statistical analyses. Paired Student’s t-test was performed to examine statistical significance (expressed as mean ± SEM or SD).

## Results

### (±)-RPE65-61 inhibits retinol isomerase activity in vitro

**Fig 2:**
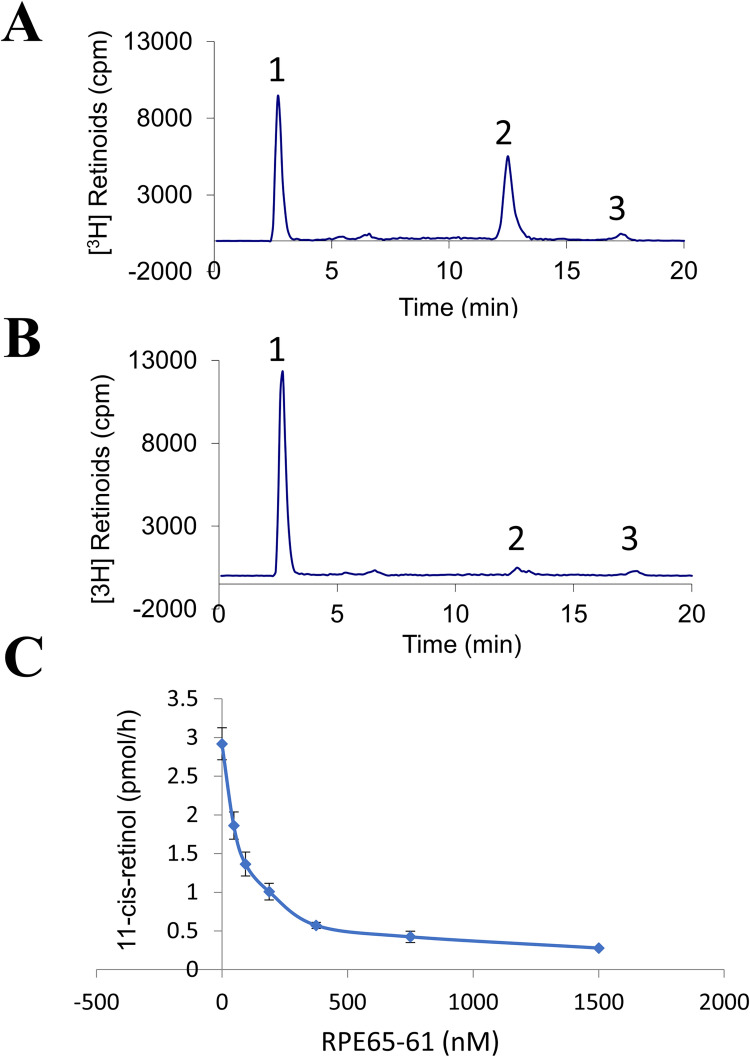
(±)-RPE65-61 inhibits retinol isomerase activity in vitro. Bovine RPE microsomes (25 μg) were incubated with 0.2 μM of all-*trans*- [^3^H]-retinol in the presence or absence of (±)-**RPE65-61** for 1h at 37°C. The generated retinoids were analyzed by HPLC coupled with flow scintillation analyzer. (A) HPLC elution profile without inhibitor; (B) with 375 nM of RPE65-61. Peak 1, retinyl esters; 2, 11-*cis*-retinol; 3 all-*trans*-retinol. (C) (±)-**RPE65-61** concentration-dependent inhibition of 11-*cis*-[^3^H]-retinol generation (mean ± SEM, n = 3).

The retinoid isomerase reaction in the RPE, the key and limiting step of the visual cycle, is catalyzed by the RPE65 protein [8–10]. Although all-*trans*-retinyl ester is the direct substrate of the isomerase [24], the almost complete insolubility of retinyl ester in water prevents its use in the isomerase assay. Therefore, all-*trans*-[^3^H]-retinol was utilized to produce retinyl esters in the bovine RPE microsomes. Retinyl ester formation is catalyzed by lecithin retinol acyl transferase (LRAT) localized in RPE microsomes. The retinyl esters in RPE microsomes are then converted to 11-*cis*-retinol by the bovine RPE65. We utilized bovine RPE microsomes and performed an in vitro isomerase test with and without the inhibitor to see if (±)-**RPE65-61** can suppress retinol isomerase activity. The retinoids were extracted by methanol and hexane and quantified using HPLC (Fig 2A–2C). All-*trans*-[^3^H]-retinol incubation with RPE microsomes resulted in retinyl esters and 11-*cis*-retinol, as predicted (Fig 2A). The addition of 375 nM of (±)-**RPE65-61** to the isomerase reaction resulted in a significant decrease of 11-*cis*-retinol production (peak 2) (Fig 2B). (±)-**RPE65-61** did not suppress the production of retinyl ester (peak 1), implying that LRAT was not inhibited. (±)-**RPE65-61** also reduced isomerase activity with an apparent IC_50_ of 80 nM in a concentration-dependent manner (Fig 2C). Despite relatively low aqueous solubility of (±)-**RPE65-61** (188 μM), it was possible to test the compound’s inhibitory action at doses up to 1500 nM (Fig 2C). In a binding experiment assessing interaction with Retinol-Binding Protein 4 (RBP4), the general selectivity of (±)-**RPE65-61** binding for RPE65 was evaluated. General selectivity of (±)-**RPE65-61** binding for RPE65 was assessed in a binding assay measuring compound interaction with Retinol-Binding Protein 4 (RBP4). Consistent with high RPE65 specificity, (±)-RPE65-61 did not show activity in the RBP4 binding assay (data not shown).

### (±)-RPE65-61 confers an uncompetitive inhibition of the isomerase activity of RPE65

**Fig 3:**
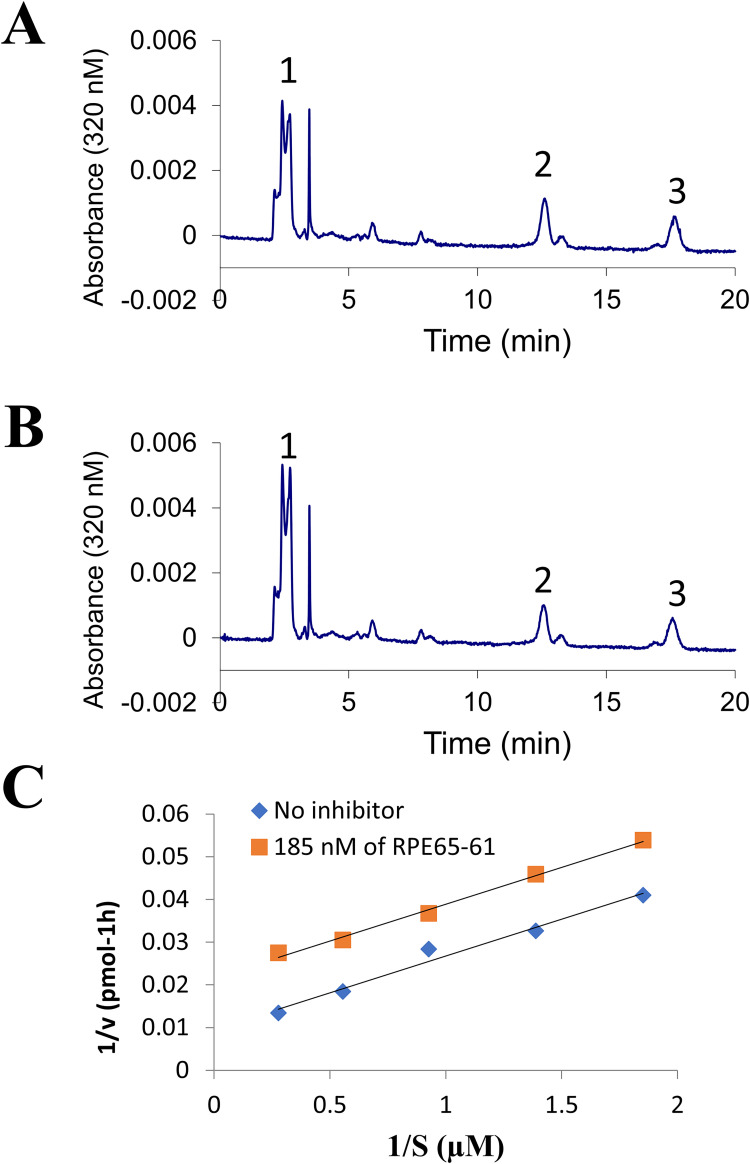
Uncompetitive inhibition of RPE65 isomerase by (±)-RPE65-61 in the liposome-based assay. All-*trans*-retinyl ester incorporated in liposomes was used as a substrate for purified RPE65 in the isomerase assay. (A). HPLC elution profile of the reaction products without inhibitor. (B) with 180 nM of (±)-**RPE65-61**; Peak 1, retinyl esters; 2, 11-*cis*-retinol; 3, all-*trans*-retinol. (C) Lineweaver-Burk plot of 11-*cis*-retinol generated by RPE65. Liposomes with increasing concentrations (S) of all-*trans*-retinyl palmitate were incubated with 25 μg of purified chicken RPE65 in the absence (♦) or in the presence of (±)-RPE65-61 (180 nM) (■).

Next, we evaluated the inhibition of RPE65 in a one-step reaction using its direct substrate retinyl ester to rule out the possibility that decreased 11-*cis*-retinol synthesis was caused by the inhibition of LRAT activity and to explore the manner of inhibition of RPE65 by (±)-**RPE65-61**. We chose recombinant chicken RPE65 in this test because it has a greater isomerase activity than RPE65 from rod dominant species. We used a liposome-based isomerohydrolase test since retinyl ester is insoluble in water; all-*trans*-retinyl palmitate, the RPE65 substrate, was integrated into liposomes (1,2-dioleoyl-sn-glycero-3-phosphocholin: 1,2-dilauroyl-sn-glycero-3-phosphocholine), 85:15) [25], and purified chicken RPE65 was treated with different quantities of the liposome-containing substrate with or without (±)-**RPE65-61**. (±)-**RPE65-61** substantially decreased both Vm and Km, as seen in the graph, resulting in a plot with two parallel lines, typical for uncompetitive inhibition [26] (Fig 3A–3C). According to the formula for uncompetitive inhibition, (±)-RPE65-61 has a Ki of 119 ± 11 nM. Taken together, this demonstrated that RPE65-61 is an uncompetitive inhibitor of RPE65.

### Systemic injection of (±)-RPE65-61 inhibits 11-cis-retinal chromophore regeneration following bleaching in mice

**Fig 4:**
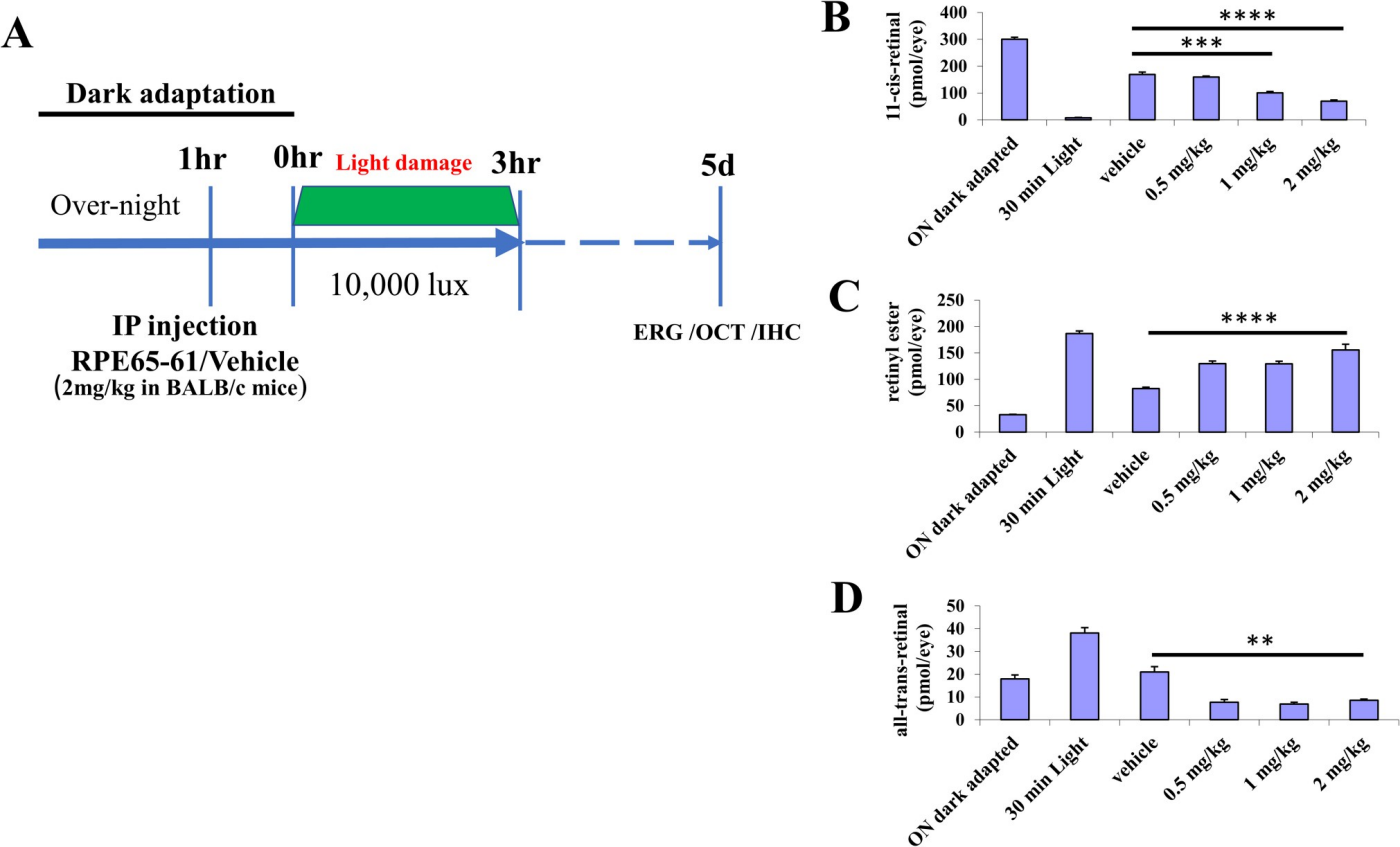
Systemic treatment by inhibitor (±)-RPE65-61 delays chromophore regeneration in BALB/cJ mice. (A) Schematic representation of the experimental design for the study of (±)-**RPE65-61** retina protection against light damage in vivo. (B-D) Retinoids levels in the inhibitor treated mouse eyes during dark adaptation after the photobleach. Dark-adapted mice were injected with varying amounts of (±)-**RPE65-61**, and subjected to light-adaptation under 5000 lx of fluorescent light for 30 min. The eyes were harvested for HPLC retinoid profiling after 30 min of dark-adaptation, to measure levels of (B) 11-*cis*-retinal, (C) retinyl ester, and (D) all-*trans*-retinal. Student’s t-test. ** P< 0.01, *** < 0.001 and **** < 0.0001. (Mean ± SEM, n = 8).

We administered (±)-**RPE65-61** into BALB/cJ mice intraperitoneally to measure its effect on visual chromophore regeneration following illumination with 5,000 lux fluorescent light for 30 minutes. After 30 minutes of light exposure, mice were returned into the dark for 30 minutes to regenerate the visual chromophore before harvesting the eyeballs. Retinoids from the collected eyes were extracted under low red light, and the levels of 11-*cis*-retinal, all-*trans*-retinal, and all-*trans*-retinyl ester were measured by HPLC (Fig 4B–4D). (±)-**RPE65-61** inhibited chromophore regeneration in a dose-dependent manner, with animals injected with 2 mg/kg of (±)-**RPE65-61** showing the most considerable reduction in 11-*cis*-retinal regeneration (40 percent of vehicle control). Moreover, animals injected with 0.5, 1, and 2 mg/kg of (±)-**RPE65-61** had higher levels of all-*trans*-retinyl ester, RPE65’s substrate, indicating reduced RPE65 activity. In addition, all-*trans*-retinal levels generated by photoisomerization of 11-*cis*-retinal were also reduced in the presence of the inhibitor. Since most of the 11-*cis*-retinal in the mouse eye is bound to opsin, the measured 11-*cis*-retinal equals rhodopsin [32].

Collectively, these data suggest that (±)-**RPE65-61** injection reduces 11-*cis*-retinal and rhodopsin regeneration in the mouse eye. Based on these dosages’ tests, we designed the experiment for light damage protection.

### (±)-RPE65-61 treatment prevents declines of retinal thickness induced by light damage

**Fig 5:**
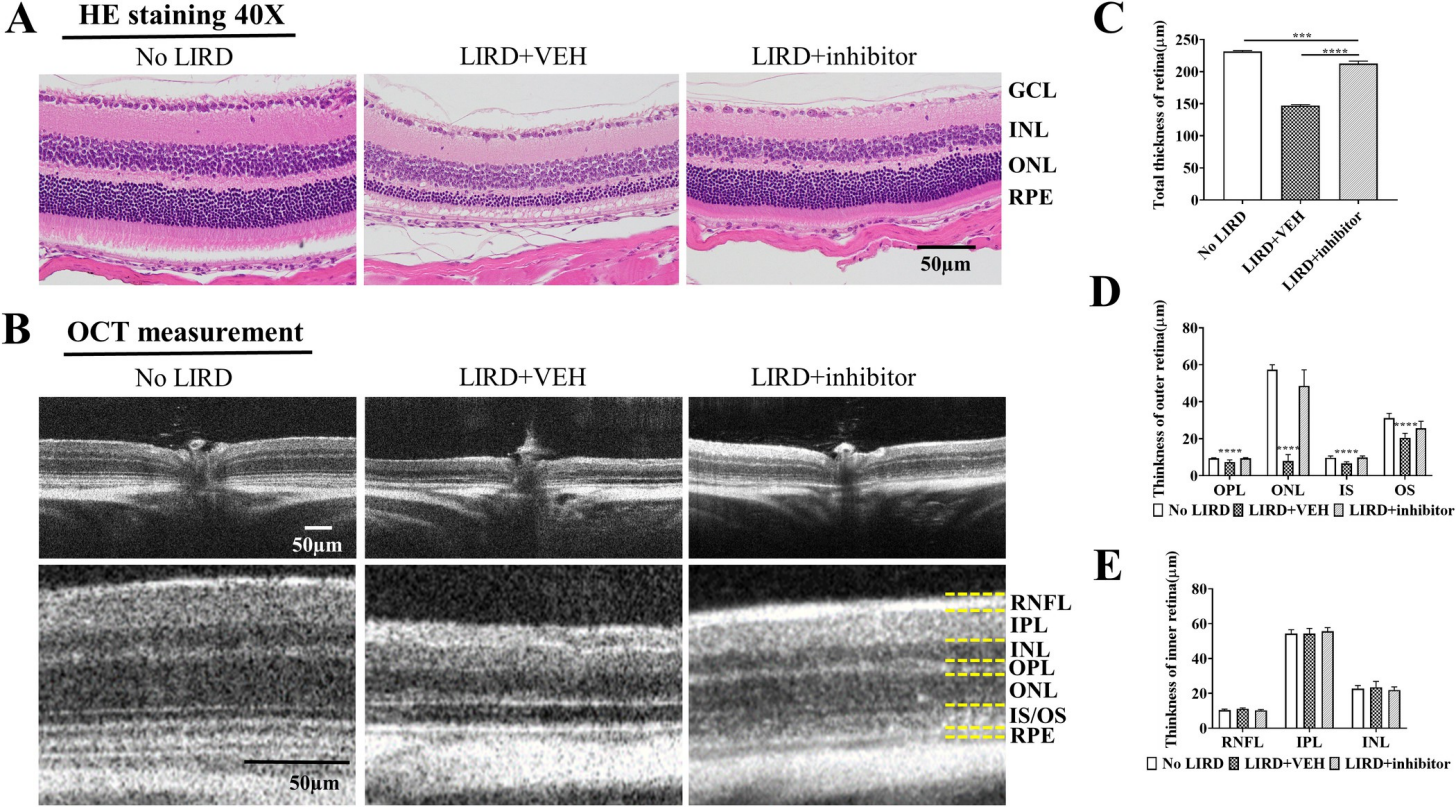
Effect of (±)-RPE65-61 on protection against retinal degeneration. LIRD caused the degeneration in the retina and RPE, which was rescued by inhibitor at the dose of 2 mg/kg in BALB/c mice. (A) Representative histological analysis for the retinas of (±)-**RPE65-61** injected mice 5 days post-LIRD. Retinal cross-sections from three groups were stained with H&E for morphological comparison, 40× magnification. Scale bar, 50μm. (B) Representative OCT images of mice treated with RPE65-61. (C-E) Quantification of the protective effects of (±)-RPE65-61 from OCT measurements are shown by measuring the average total thickness of retina (C), outer retina (D) and inner retina (E). RPE: retinal pigment epithelium; IS: outer segment; OS: outer segment; ONL: outer nuclear layer; OPN: outer plexiform layer; INL: inner nuclear layer; IPN: inner plexiform layer; RNFL: The retinal nerve fiber layer. The three groups: No LIRD control, LIRD + Vehicle or LIRD + (±)-**RPE65-61**. Data expressed as mean ± SEM. n = 8 per group. Student’s t-test. *P < 0.05, ** < 0.01 and *** < 0.001, **** < 0.0001.

Light-induced retinal damage (LIRD) is a good model for studying different forms of retinal degeneration. Functioning rhodopsin is needed for the harmful impact of light [33]; therefore, the decrease of 11-*cis*-retinal chromophore through inhibition of the visual cycle may prevent LIRD [2, 34]. We examined whether (±)-**RPE65-61** can have a neuroprotective impact on the retina in the LIRD model based on its selective suppression of RPE65 by (±)-**RPE65-61**. To see if this was true, completely dark-adapted mice were given either (±)-**RPE65-61** (2 mg/kg, IP) or vehicle 1 hour before LIRD (10,000 lux for 3 hr) (Fig 4A). Five days after LIRD, thickness of the total retina (Fig 5A–5C) and that of different retinal layers (Fig 5D and 5E) were measured using optical coherence tomography (OCT). We found that LIRD decreased retinal thickness, while (±)-**RPE65-61** attenuated the decline of retinal thickness in LIRD mice compared to vehicle-treated mice, suggesting a protective effect against degeneration of the retina. The protective effect of (±)-**RPE65-61** on retinal thinning in LIRD model is attributed to the compound’s suppression of the visual cycle.

### Suppressing RPE65 activity preserves photoreceptors in LIRD mice

**Fig 6:**
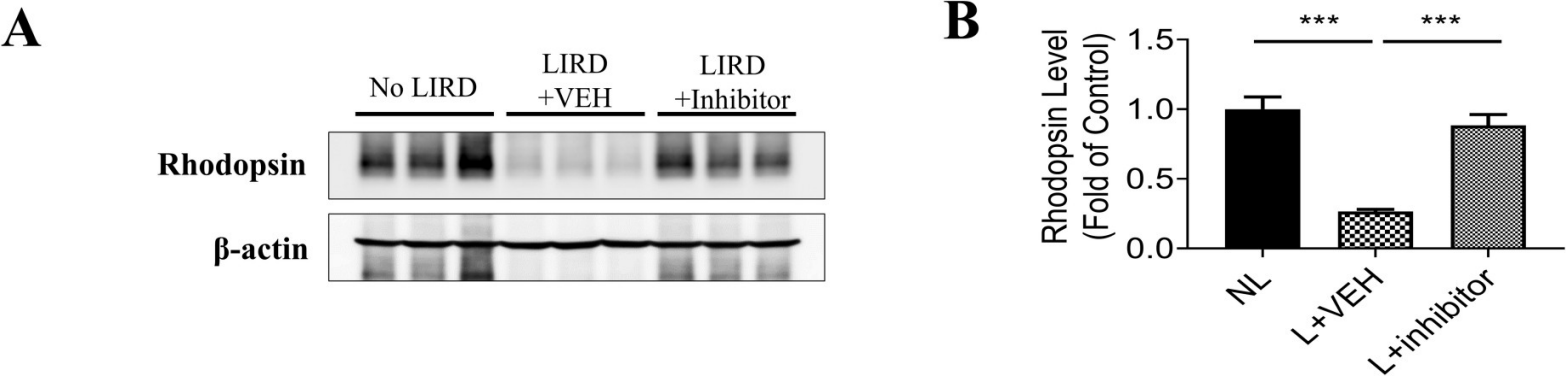
Analysis of rhodopsin level in LIRD mice treated with (±)-RPE65-61. LIRD caused the degeneration in the retinal photoreceptor cells and decrease of rhodopsin levels, which was preserved by inhibitor at the dose of 2 mg/kg in BALB/c mice. (A) Expression levels of opsin in the retinas of control and LIRD mice were measured using anti-rhodopsin (1D4) antibody. (B) Opsin monomer level was analyzed by densitometry and normalized by β-actin level. Data expressed as mean ± SEM. n = 4 per group. Student’s t-test. *P < 0.05, ** < 0.01.

The decrease of the retinal thickness of LIRD mice suggests that rod photoreceptors degenerate, which might change photoreceptors’ proteins expression. We performed immunoblot analysis of rhodopsin in retinal lysates from LIRD mice (Fig 6A and 6B). Rhodopsin signal in the blots normalized to beta-actin levels revealed reduced levels of rod opsin protein in LIRD mice treated with vehicle, relative to normal controls. Mice injected with (±)-**RPE65-61** before damaging light exposure had substantially greater rod opsin levels than LIRD animals treated with vehicle, indicating that RPE65 inhibition with (±)-**RPE65-61** prevented light-induced rod photoreceptor degeneration (Fig 6A and 6B). These results demonstrate a protective role of (±)-**RPE65-61** against photoreceptors degeneration in LIRD mice.

### Assessment of LIRD mice retinal function by electroretinography (ERG)

**Fig 7:**
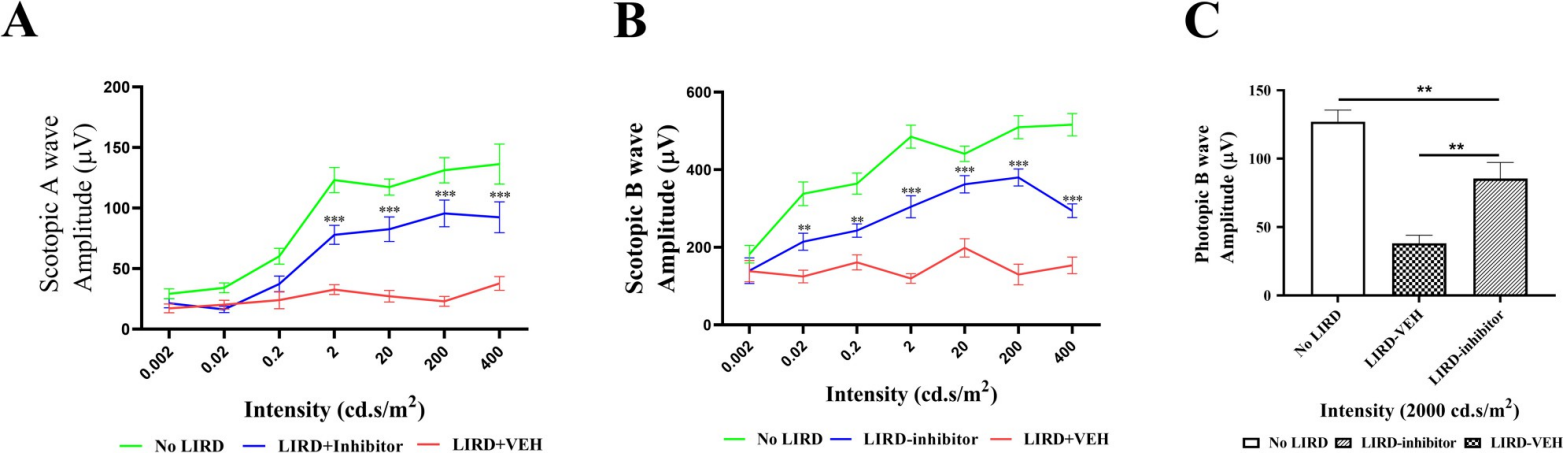
ERG responses of (±)-RPE65-61 treated LIRD mice. Retina photoreceptor function was measured by ERG 5 days after light exposure. (A) Comparison of averaged scotopic a-wave amplitudes from three mouse groups. Seven different stimulus intensities were used, ranging from 0.002 to 400 cd s/m2. (B) Comparison of the averaged scotopic b-wave amplitude from the three groups. (C) Comparison of the averaged photopic b-wave amplitude from the three groups: No LIRD control, LIRD + Vehicle or LIRD + (±)-**RPE65-61**, Student’s t-test was used (n = 8, mean ± SEM, *P < 0.05, ** < 0.01 and *** < 0.001).

To evaluate retina function after light damage, we measured ERG responses of (±)-**RPE65-61** injected mice five days post-LIRD. Dependences of scotopic a-wave amplitudes and b-wave amplitudes on light stimulus are shown for three mouse groups in Fig 7A and 7B. Seven different stimulus intensities, ranging from 0.002 to 400 cd s/m2, were employed in this study. Significantly higher scotopic a-wave and b-wave amplitudes were observed in the LIRD mice with the inhibitor treatment (Fig 7A and 7B). Furthermore, considerably stronger photopic responses from (±)-**RPE65-61** injected animals with LIRD than the vehicle-treated control implies that (±)-**RPE65-61** protects cone photoreceptors as well. The data presented here collectively demonstrate that slowing of the visual cycle with (±)-**RPE65-61** provides a neuroprotective effect on the retina exposed to the LIRD.

### Detection of the cGAS-STING/inflammation/apoptosis signal pathway

**Fig 8:**
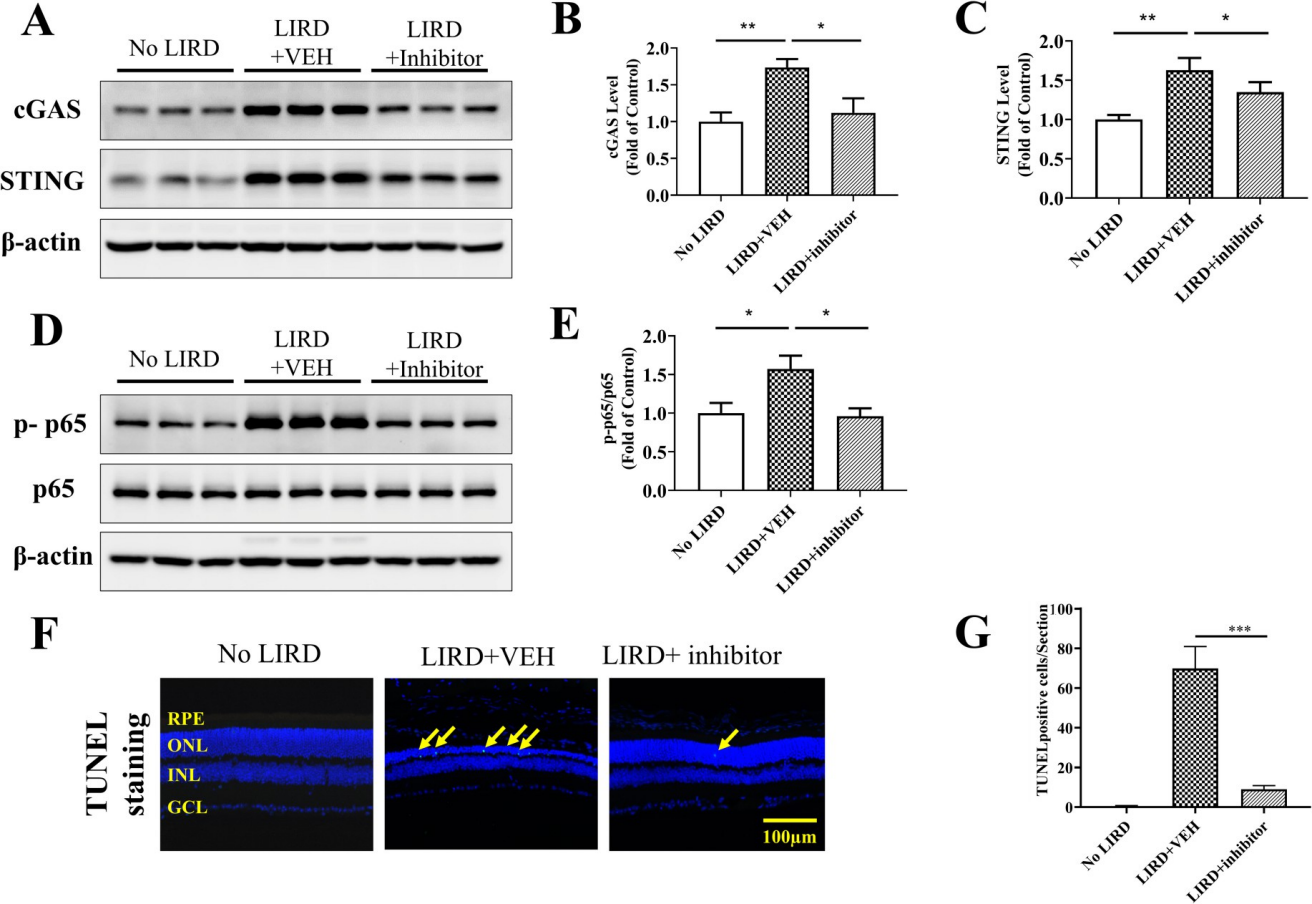
Analyses of cGAS-STING pathway and retina cells apoptosis in LIRD mice treated with (±)-RPE65-61. (A-C) cGAS and STING protein levels in retina were measured by Western blot analysis with β-actin as loading control and densitometry quantification. (D, E) Levels of phosphorylated NF-κB (p-p65) were determined by Western blotting. Total NF-κB (p65) was used as an internal control and for densitometry quantification. Each lane represents an individual mouse. (F, G) Photoreceptor cell apoptosis was evaluated by TUNEL staining on the retinal sections at the dose of 2 mg/kg in BALB/c mice and correlating quantitative analysis. 20X magnification, Scale bar, 100μm. Student’s t-test was used (n = 5, mean ± SEM, *P < 0.05, ** < 0.01).

Our results showed that a single dose of 2 mg/kg (±)-**RPE65-61** protected retinal structure and function in LIRD mouse model. It is known that strong light may cause DNA damage and even double-stranded DNA breaks [35]. The cGAS–STING pathway induced by cytoplasmic DNA release can activate type I interferons (IFNs) and other inflammatory cytokines. To further explore the mechanism of (±)-**RPE65-61** protection from LIRD, we examined the expression of the genes involved in the cGAS-STING pathway. Expression of the cGAS and STING in the retina was upregulated by LIRD but was suppressed by (±)-**RPE65-61** (Fig 8A–8C) and upregulation of phosphorylated nuclear factor κ B p65 subunit (p-p65) (Fig 8D and 8E), associated with LIRD was mitigated by (±)-RPE65-61 treatment. To evaluate the protective effect of (±)-RPE65-61 against LIRD apoptosis, we analyzed cell death in the retinas of LIRD mice using the terminal deoxynucleotidyltransferase dUTP nick-end labeling (TUNEL) method. Retinal section analysis revealed a large increase in the numbers of TUNEL-positive photoreceptor cells in the vehicle injected group. A number of TUNEL positive cells and enhanced apoptosis were significantly decreased by (±)-**RPE65-61** treatment (Fig 8F and 8G).

## Discussion

The present study aimed to evaluate the partial suppression of the visual cycle as an instrument to preserve retinal structure and function in an animal model of light-induced retina degeneration. The goal was to develop a small non-retinoid RPE65 inhibitor and to determine its retina-protective effect on LIRD. The development of safe and effective small-molecule therapeutics for blinding retinal degenerative diseases remains a significant challenge. The present study demonstrates that (±)-**RPE65-61**, a novel non-retinoid compound, effectively and selectively inhibits RPE65, a key enzyme of the visual cycle. Further, (±)-**RPE65-61** also protects retina structure and function from LIRD, suggesting a therapeutic potential for retinal degeneration.

The visual cycle is a sequence of biochemical events that begin when a photon of light interacts with the visual pigment protein rhodopsin, resulting in an electrophysiological signal and visual perception. The process proceeds with a number of reactions that lead to the rhodopsin molecule regeneration. The chromophore, 11-*cis*-retinal, is synthesized first, and several retinoid metabolizing enzymes and retinoid-binding proteins are involved in the regeneration of the rhodopsin. Experimental animal models of retina diseases suggest that an all-*trans*-retinal, toxic byproduct of the visual cycle, must be effectively cleared from the retina for maintaining retinal health. It has been shown that the loss of clearance mechanism caused by mutations in ABCA4, a gene of the ATP-binding cassette transmembrane protein, which eliminates all-*trans*-retinal from rod photoreceptors, causes retina degeneration in STGD. All-*trans*-retinal is toxic itself and can react with membrane lipids to form toxic bis-retinoids. Findings from Boyer et al. [14] support the idea that bis-retinoid can be generated in vivo from 11-*cis*-retinal. Under light conditions, the relative contributions of 11-*cis*- and all-*trans*-retinal to bis-retinoid generation are unknown, however only a small amount of 11-*cis*-retinal is in free aldehyde form in the retina and may react with lipids and it is likely that all-*trans*-retinal is the major origin of A2E. Functional declines of the retinas were observed in *Abca4−/−/Rdh8−/−* animals, along with retinal degeneration, which was linked to an aberrant buildup of bis-retinoids [36, 37]. All-*trans*-retinal clearance delay and, consequently, abnormal accumulation of bis-retinoids may also result from aging and cause dry AMD. DNA damage, oxidative stress, complement activation, and mitochondrial dysfunction have all been shown to be associated with aberrant accumulation of bis-retinoid condensation products in both the retina and the RPE [38, 39].

A key question now is how to reduce the generation of toxic byproducts of the visual cycle. To further explore retinoid dynamics in visual function, particularly in pathological states, it is critical to discover targetable molecules, proteins, and pathway(s) and eventually create reasonable treatment methods to avoid visual deterioration. One of the essential biochemical steps in the visual cycle is the conversion of all-*trans*-retinyl ester to 11-*cis*-retinol by RPE65. RPE65 is an ideal modulator because it is a rate-limiting enzyme of the visual cycle and is nearly entirely expressed in the RPE [18, 40, 41]. After three months of oral treatment, emixustat, a non-retinoid RPE65 inhibitor, showed a dose-dependent decrease in A2E levels in *Abca4−/−* mice [42]. Furthermore, *Rpe65−/−* animals, who lack rhodopsin in photoreceptors, are resistant to LIRD, as are mice and rats treated with RPE65 inhibitors [27, 33, 34]. Hence, the manipulation of the visual cycle gives significant insights towards creating possible retinal degeneration therapy applications.

Conceptually, inhibition of the visual cycle can experimentally prevent retinal degeneration by reducing all-*trans*-retinal and its lipid condensation product toxicity. Partially blocking the visual cycle might reduce the synthesis of harmful all-*trans*-retinal and its conjugation product A2E by slowing rhodopsin renewal through the visual cycle. A low dosage of a potent inhibitor is ideal for this mechanism of action. While visual cycle inhibition has the potential to improve disease processes, its modes of action make it inherently vulnerable to side effects. Nyctalopia and dyschromatopsia are direct extensions of visual cycle suppression, as revealed in emixustat clinical studies [43, 44]. Emixustat is a potent RPE65 inhibitor with IC_50_ values of 4 nM, 150 nM, and 232 nM as shown by in vitro retinol isomerase assay by different authors [41, 42, 45, 46]. Consistent with the mechanism of action, the emixustat drastically and dose-dependently reduced the ERG b-wave recovery after photobleaching in mice [42]. Clinically, a single oral dose of emixustat at 2–75 mg/day in a Phase 1a trial was found to induce a dose-dependent reduction in the rate of rod function recovery after photobleaching with suppression of the rod ERG b-wave amplitude starting from the dose 40 mg [44]. Subjectively, a single dose induced no visual adverse effects (AEs) in the 2–10 mg cohorts while showing the dose-dependent increase in AEs reaching 100% at doses higher than 40 mg [44, 47]. In a Phase 1b trial, emixustat was dosed for 14 days at 5–40 mg/day. All subjects in this study who received doses of 20 mg and higher experienced visual AEs. An important conclusion from these Phase 1a and 1b studies is that doses of emixustat required to achieve the desired effect of ~50% suppression of the rod ERG b-wave and clinical efficacy [47] also induced mechanism-based AEs in 100% of participants [43]. In a Phase 2 trial with dry AMD patients, low doses (2–10 mg) induced ocular AEs in 93% of drug-treated dry AMD patients [22]. In a subsequent Phase 2b/3 clinical trial, emixustat at low 2.5–10 mg doses failed to slow the rate of the atrophy progression in dry AMD patients [23]. The incidence of ocular AEs was 57% in treatment groups, and dropout rates in the emixustat arms were doubled over the placebo group, which were driven by mechanism-based AEs [23]. A Phase 3 clinical trial of the 10 mg emixustat dose in patients with STGD1 is ongoing. Overall, the clinical data indicate that emixustat cannot be administered at efficacious doses without inducing the unacceptable level of mechanism-based AEs. There are a number of other serious emixustat liabilities, such as a pharmacokinetics/pharmacodynamics (PK/PD) disconnect in humans. The PD response in the retina and signs of retinal toxicity were evident several days after administration of a single oral dose [44] which was inconsistent with the systemic PK where emixustat showed extremely fast metabolism and high clearance [43]. No detectable plasma levels were seen after administration of the 2 mg dose [44], and systemic exposure was very low even at the highest dose (40 mg) [43]. Similarly, emixustat metabolites are cleared from the circulation very quickly [48]. This prolonged PD response may indicate drug accumulation in the retina. Emixustat is a substrate for RPE LRAT that can reversibly palmitoylate the drug in the RPE to form emixustat palmitamide [46]. Emixustat palmitamide may represent a depot form of the drug in the retina. The conversion to emixustat palmitamide by LRAT is a negative attribute as it is likely to be responsible for the PK/PD mismatch and may be responsible for the long duration of ocular AEs after a single dose administration.

One other issue revealed for emixustat is that its clearance in humans relies on the unusual route of metabolism driven involving oxidative deamination by Vascular Adhesion Protein-1 (VAP-1), a circulating amine oxidase [48]. The drug also acts as an inhibitor of its metabolizing enzyme, VAP-1 [48], which raises drug-drug interactions (DDI) concerns for therapeutics (e.g., amlodipine) that are also metabolized by VAP-1. Furthermore, our own data (not shown) indicates that emixustat is a cytochrome P-450 subtype 2C19 (CYP2C19) inhibitor, which raises an additional DDI concern. Primary amine substrates that inhibit VAP-1 act as irreversible inhibitors that covalently bind to the enzyme’s active site [49]. Emixustat is likely to bind covalently to VAP-1. Covalent inhibitors can generate immunogenic protein adducts, which may induce a deadly idiosyncratic response [50]. Overall, RPE65 is a highly attractive drug target for therapeutic intervention in STGD1. However, the most advanced RPE65 inhibitor, emixustat, may be less than optimal for testing the hypothesis on clinical efficacy of RPE65 inhibitors as a STGD1 therapy due to unavoidable mechanism-based AEs and suboptimal drug-like properties. Thus, our goal is to develop a novel and orally bioavailable RPE65 inhibitor that lacks emixustat liabilities such as LRAT specificity, unconventional route of metabolism by VAP-1, and CYP2C19 and VAP-1 inhibition.

Thus, we initially designed racemic sulfoximine (±)-**RPE65-61**, a novel compound derived from emixustat that exhibits significant RPE65 inhibition (IC_50_ = 80 nM) and presents a suitable hit with which to initiate a medicinal chemistry hit-to-lead campaign. The sulfoximine can serve as a bioisostere for a variety of functional groups, and the use of it has increased substantially in recent years, as evidenced by the development of synthetic methods, composition of matter patents, and clinical entries. The use of this functional group in our rational drug design efforts was made for the following reasons; (**1**) the sulfoximine NH provides a hydrogen bond donating group, which was anticipated to participate in an H-bond interaction with Thr147 in a similar manner as the emixustat hydroxyl group; (**2**) the electron withdrawing capability of the sulfoximine inductively lowers the pKa of the pendant primary amine (calculated (±)-**RPE65-61** pKa = 8.11, emixustat pKa = 9.88, ChemDraw), which could potentially mitigate risks for i) potent binding at human ether-a-go-go K+ channel (hERG) and potential cardiovascular AEs, ii) poor passive permeability and/or active transport-mediated efflux (i.e., P-glycoprotein transporter, breast cancer resistance protein, multidrug resistance-associated protein etc.), iii) lysosomal accumulation and phospholipidosis, and iv) reduced nucleophilicity of the primary amine due to the sulfoximine may potentially reduce activity of LRAT; (**3**) the sulfoximine nitrogen provides an additional vector with which to probe unexplored RPE65 structure-activity relationship (SAR); (**4**) the polarity of the sulfoximine allows for modulation of lipophilicity, which can help optimize physicochemical and absorption distribution metabolism excretion properties; (**5**) the sulfoximine provides novel intellectual property. Here we have demonstrated that (±)-RPE65-61 efficiently and selectively inhibited RPE65 catalyzed reaction *in vitro*, however the mechanism of RPE65 inhibition by (±)-**RPE65-61** is currently unclear. (±)-**RPE65-61** can bind RPE65 at its active site, competing with the binding of a retinyl ester substrate. It may also bind to the enzyme-substrate complex causing uncompetitive inhibition. To discriminate between these two alternatives, we studied (±)-**RPE65-61** inhibition using liposome-based isomerase assay with recombinant RPE65. (±)-**RPE65-61** inhibited isomerase reaction uncompetitively, suggesting that this inhibitor does not bind to the free RPE65, and it rather binds to the enzyme-substrate complex [26]. A similar pattern of the RPE65 inhibition was observed previously for the α-phenyl-N-tert-butylnitrone inhibitor [34]. To clarify the mode of binding of (±)-**RPE65-61** to the RPE65-substrate complex, future work on the crystal structure of the RPE65-inhibitor complex is necessary.

(±)-**RPE65-61** was also tested in vivo to see whether it might inhibit RPE65. The HPLC retinoid profile analysis revealed that animals injected with (±)-**RPE65-61** had a dose-dependent delay in chromophore regeneration following photobleach, indicating a slower visual cycle. (±)-**RPE65-61** inhibited 11-*cis*-retinal regeneration after photobleaching with a higher potency than another non-retinoid CU-239 described previously [27]. This effect correlates with significantly lower IC_50_ for (±)-**RPE65-61** as compared to CU239 in vitro RPE65 inhibition. As expected, mice treated with (±)-**RPE65-61** accumulate higher levels of retinyl esters because retinyl esters are converted to 11-*cis*-retinol slower in the presence of the inhibitor.

Furthermore, we administered 2 mg/kg (±)-**RPE65-61** to mice to see if visual cycle suppression induced by the compound might protect the retina from light-induced retinal damage. Our post-LIRD histological and functional studies of the retina using ERG revealed that (±)-RPE65-61 protects against LIRD, as predicted.

The mechanisms of retina protection from light damage through RPE65 inhibition are currently unclear. It is known that intense light causes DNA breaks [35], and all-*trans*-retinal mediated this process [51]. It has been reported that cGAS-STING is a sensor of DNA damage [52]. This work shows that (±)-**RPE65-61** inhibition protected photoreceptor cells from the activation of cGAS-STING signal pathway, possibly through the inhibition of visual cycle and, consequently, through decrease of DNA light damage, in an acute mouse model of AMD, accompanied by suppressing the upregulation of the genes involved in apoptosis and inflammatory responses.

In conclusion, (±)-**RPE65-61**, a new non-retinoid molecule, is a selective and strong inhibitor of RPE65, according to our findings. Systemic administration of (±)-**RPE65-61** inhibitor in the acute LIRD model partially preserves photoreceptors’ structure and function. We have shown that inhibition of RPE65 down-regulates cGAS-STING pathway, decreases inflammation, reduces apoptosis in the eye and that (±)-**RPE65-61** is neuroprotective and preserves the visual function of LIRD mice. Thus, we concluded that (±)-**RPE65-61** is a potent visual cycle modulator and likely possesses a promising therapeutic potential for retinal degeneration. As VAP-1 oxidation is the major metabolic pathway for emixustat, our ongoing drug design efforts primarily focus on novel analogs that alter the amine linker of (±)-**RPE65-61** in order to prevent oxidation alpha to the primary amine. We are currently exploring reported medicinal chemistry methods used to reduce cytochrome-, monoamine oxidase-, and aldehyde oxidase-mediated oxidations of similar aza-containing systems. This includes deuterium atom incorporation, utilization of groups that may help block metabolism via steric hindrance, altering the linker shape (i.e., via introduction of a ring system), or by modulating the electronics at the site of metabolism [53]. In addition to blocking VAP-1 oxidation, some of our modifications are also designed to hinder LRAT palmitoylation, and CYP2C19 and VAP-1 inhibition. Lastly, we will also resolve (±)-**RPE65-61** to determine if there is an enantiopreference for inhibition of RPE65. The PK study (required to establish adequate exposure before performing in vivo efficacy experiments) will be conducted. Preliminary *in vivo* bioavailability and retinal exposures will be assessed for a limited set of optimized molecules through upcoming *in vivo* PK-PD and mouse *Abca4*^-/-^ and *Abca4*^*-/-*^*Rdh8*^*-/-*^ efficacy studies. These data will be reported in due course.

## Supporting information

S1 Raw images Original uncropped and unadjusted blots corresponding to Figs 6A, 8A and 8D from the main text. Molecular size markers are shown.
(TIF)
