# Supplementary figures and images for: The novel visual cycle inhibitor (±)-RPE65-61 protects retinal photoreceptors from light-induced degeneration
Source: PLoS One. 2022 Oct 13;17(10):e0269437. doi: 10.1371/journal.pone.0269437 (PMC9560169; doi:10.1371/journal.pone.0269437)

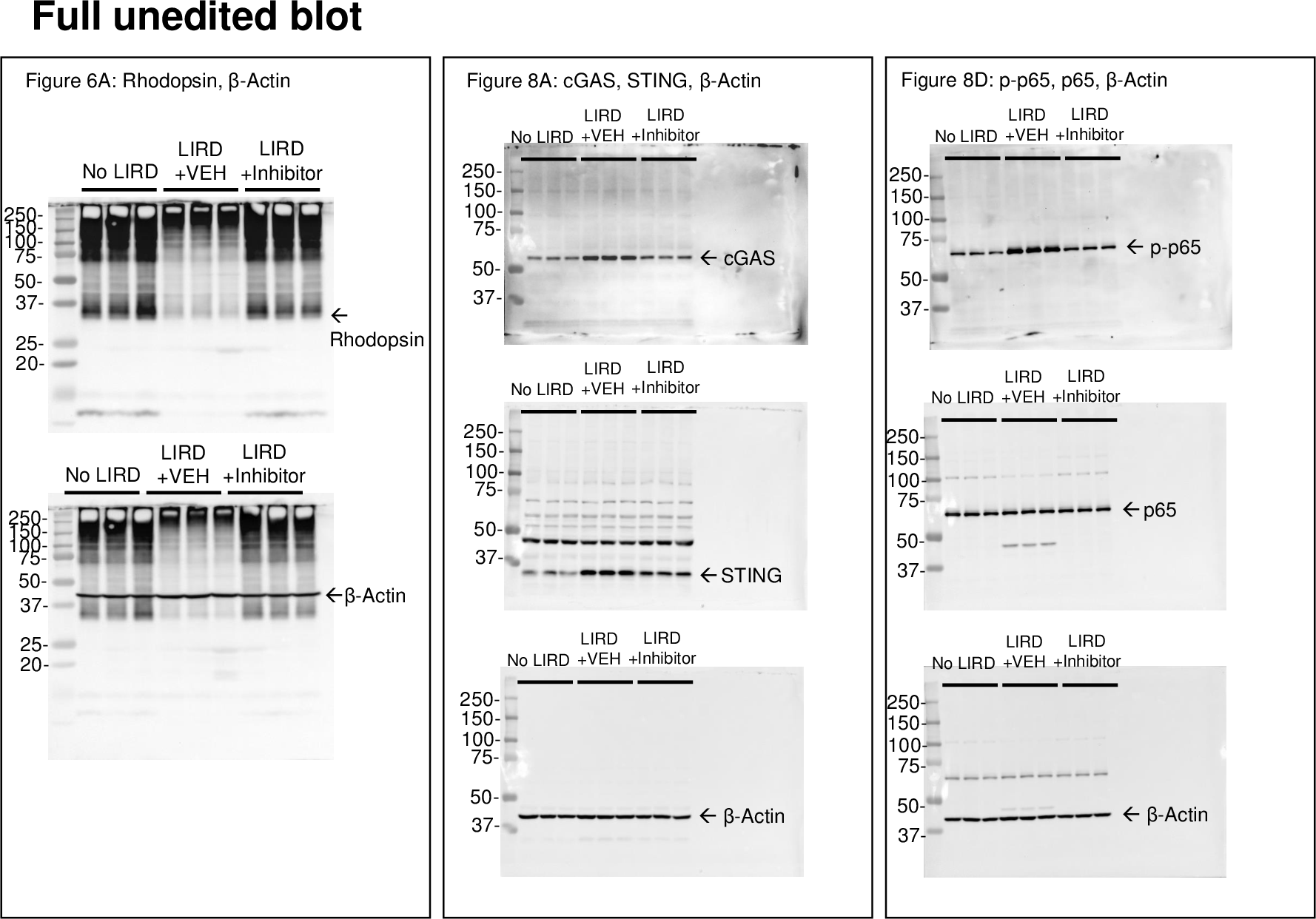

Supplement: S1 Raw images — Molecular size markers are shown. (TIF) [file pone.0269437.s001.tif]
